# Multi‐institutional, prospective, randomized, double‐blind, placebo‐controlled phase IIb trial of the tumor lysate, particle‐loaded, dendritic cell (TLPLDC) vaccine to prevent recurrence in high‐risk melanoma patients: A subgroup analysis

**DOI:** 10.1002/cam4.3969

**Published:** 2021-05-12

**Authors:** Robert C. Chick, Mark B. Faries, Diane F. Hale, Phillip M Kemp Bohan, Annelies T. Hickerson, Timothy J. Vreeland, John W. Myers, Jessica L. Cindass, Tommy A. Brown, John Hyngstrom, Adam C. Berger, James W. Jakub, Jeffrey J. Sussman, Montaser Shaheen, Guy T. Clifton, Hyohyun Park, Amanda J. Sloan, Thomas Wagner, George E. Peoples

**Affiliations:** ^1^ Brooke Army Medical Center Fort Sam Houston TX USA; ^2^ The Angeles Clinic Santa Monica CA USA; ^3^ Huntsman Cancer Institute University of Utah Salt Lake City UT USA; ^4^ Rutgers Cancer Institute of New Jersey New Brunswick NJ USA; ^5^ Mayo Clinic Rochester MN USA; ^6^ University of Cincinnati Cincinnati OH USA; ^7^ University of Arizona Tucson AZ USA; ^8^ Orbis Health Solutions Greenville SC USA; ^9^ Cancer Vaccine Development Program San Antonio TX USA

**Keywords:** cancer vaccine, immunotherapy, melanoma, personalized medicine

## Abstract

**Background:**

Checkpoint inhibitors (CPI) in combination with cell‐based vaccines may produce synergistic antitumor immunity. The primary analysis of the randomized and blinded phase IIb trial in resected stage III/IV melanoma demonstrated TLPLDC is safe and improved 24‐month disease‐free survival (DFS) in the per treatment (PT) analysis. Here, we examine efficacy within pre‐specified and exploratory subgroups.

**Methods:**

Stage III/IV patients rendered disease‐free by surgery were randomized 2:1 to TLPLDC vaccine versus placebo. The pre‐specified PT analysis included only patients completing the primary vaccine/placebo series at 6 months. Kaplan–Meier analysis was used to compare 24‐month DFS among subgroups.

**Results:**

There were no clinicopathologic differences between subgroups except stage IV patients were more likely to receive CPI. In stage IV patients, 24‐month DFS was 43% for vaccine versus 0% for placebo (*p* = 0.098) in the ITT analysis and 73% versus 0% (*p* = 0.002) in the PT analysis. There was no significant difference in 24‐month DFS when stratified by use of immunotherapy or CPI. For patients with resected recurrent disease, 24‐month DFS was 88.9% versus 33.3% (*p* = 0.013) in the PT analysis. All benefit from vaccination was in the PT analysis; no benefit was found in patients receiving up to three doses.

**Conclusion:**

The TLPLDC vaccine improved DFS in patients completing the primary vaccine series, particularly in the resected stage IV patients. The efficacy of the TLPLDC vaccine will be confirmed in a phase III study evaluating adjuvant TLPLDC + CPI versus Placebo + CPI in resected stage IV melanoma patients.

## Graphical abstract

TLPLDC is an autologous tumor lysate vaccine made with autologous dendritic cells to prevent recurrence in patients with high‐risk resected melanoma. In this analysis, the vaccine was found to have the most benefit in stage IV resected patients and may have a synergistic effect in combination with checkpoint inhibitors.

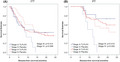

## 1 BACKGROUND

Melanoma is a common skin malignancy responsible for 55,000 deaths worldwide every year.^1^ Unlike other deadly cancers, cytotoxic chemotherapy has shown minimal benefit. Melanoma is widely accepted to be an immunogenic tumor and, as such, immunotherapy has become a primary treatment modality in advanced melanoma. Checkpoint inhibitors (CPI), beginning with ipilimumab and now including nivolumab and pembrolizumab, have significant efficacy in both the metastatic setting and as adjuvant therapy in those with node‐positive disease.^2^, ^3^, ^4^, ^5^, ^6^, ^7^, ^8^ Despite such significant advances in the medical therapy of melanoma, recurrence rates remain high among patients who present with advanced disease.^9^

Given the immunogenic nature of melanoma, cancer vaccines have also been studied widely in melanoma, but have a more varied history with minimal successes.^10^ To date, only one cancer vaccine has been FDA‐approved, and its indication is for advanced prostate cancer patients.^11^ While cancer vaccines have had minimal success, this may be related to the fact most studies have been conducted in patients with advanced, unresectable disease, where cancer vaccines are less likely to achieve a meaningful effect as monotherapy. Cell‐based vaccines may activate initial immune responses to tumors when not already present, which may be effective in patients with less advanced disease who can be rendered disease‐free by surgery. Additionally, the combination of a vaccine and CPI may extend the efficacy of immunotherapy as the initial immune response induced by vaccines can be enhanced and extended by CPIs.^12^ Such combinations are actively being studied in multiple areas, including advanced melanoma.

The tumor lysate, particle‐loaded, dendritic cell (TLPLDC) vaccine was developed as a personalized cancer vaccine to prevent recurrence in patients with resected advanced melanoma. Detailed description of the highly efficient vaccine production has been described previously and is summarized below.^13^ This multi‐center prospective, randomized, double‐blind, placebo‐controlled phase IIb trial of TLPLDC in patients with resected stage III and IV melanoma has demonstrated that TLPLDC is safe and met its primary endpoint of improved 24‐month disease‐free survival in the per treatment (PT) analysis, which included those who completed the 6‐month primary vaccine series (PVS). We have performed pre‐specified and exploratory subgroup analyses in order to better understand which patients may benefit most from this novel vaccine.

## 2 METHODS

### 2.1 Patients

Patients with completely resectable stage III/IV melanoma were identified prior to definitive surgery, then underwent standard of care (SOC) resection as indicated. After surgery, patients received systemic and/or radiation therapy at the discretion of their primary care team. Patients completed all standard of care adjuvant therapies prior to vaccination. However, when CPIs were approved for use in the adjuvant setting, beginning with ipilimumab,^2^ the protocol was amended to allow concurrent CPI and vaccination after demonstrating tolerance to the CPI after three months of therapy.

### 2.2 Randomization

Patients were randomized 2:1 to TLPLDC versus placebo. After completion of the 120th randomization, 60 additional patients were randomized 2:1 to the tumor lysate particle only (TLPO) vaccine, an alternative vaccine production strategy currently under investigation, versus TLPLDC. The additional randomized, double‐blind TLPLDC (n = 20) patients were included in the primary analysis as per the pre‐specified statistical plan. Patients were randomized after surgery to receive TLPLDC or unloaded yeast cell wall particles + dendritic cells (placebo). It was required that other systemic therapies, if indicated as determined by the treating physician, be completed prior to vaccination, except CPIs as explained above. Vaccine production occurred immediately after surgical resection and vaccine was frozen and stored for all subjects until completion of other systemic therapies when indicated.

### 2.3 Vaccine production

The vaccine was produced from a sample (1 cm^3^) of the patient's surgically resected tumor, which was lysed and loaded into yeast cell wall particles (YCWP) in a process that was described in detail previously.^13^ For DC isolation, patients received a single injection of Neupogen (G‐CSF) 300 µg (or its equivalent) subcutaneous (SQ) 24–48 hours prior to having 50–70 mL of blood collected. Patients who could not tolerate G‐CSF, or refused it, had 120 mL of blood drawn. The tumor lysate‐loaded yeast cell wall particles were then taken up by maturing DC by phagocytosis *ex vivo*; these cells constituted the vaccine product. After meeting all lot release criteria to include identity, viability, and sterility, the six vaccine doses (1–1.5 x 10^6^ TLPLDC/dose) were given through intradermal injection on the prescribed schedule of 0, 1, 2, 6, 12, and 18 months.

### 2.4 Survival analysis

The primary endpoint of the trial was 24‐month disease‐free survival (DFS), which was determined in the intention to treat (ITT) population and the pre‐specified per‐treatment (PT) population. Patients continued the vaccine series for 24‐months or until recurrence.^14^ The PT analysis excluded patients who did not complete the PVS time point of 6 months. Pre‐specified subgroup analyses included AJCC stage (III or IV),^8^ treatment with immunotherapy (yes or no), and treatment with CPI (yes or no).

An additional exploratory subgroup analysis examined whether patients who were enrolled prior to resection of primary versus recurrent disease benefited more from vaccination. For the purposes of this analysis, any patient who enrolled with recurrent disease, but a disease‐free interval less than three months, was considered to have primary disease at the time of enrollment. This was done because patients who recurred with positive nodes or metastatic disease within three months of their prior therapy were assumed to have had undetected metastases, either nodal (stage III) or distant (stage IV), at the time of their initial diagnosis. With this definition, we then compared patients with primary stage III/IV disease to those with recurrent stage III/IV disease.

The relationship between the number of doses received and clinical outcome was examined in an exploratory analysis. Overlapping subgroups of patients receiving at least 1 dose, at least 2 doses, at least 3 doses, and at least 4 doses were examined. Of note, the cohort receiving at least 4 doses is equivalent to the PT cohort.

The primary endpoint of 24‐month DFS was calculated within each subgroup using Kaplan–Meier (KM) survival analysis and survival estimates were compared using the log‐rank test at α = 0.05. Hazard ratios (HR) were calculated for each comparison using a Cox proportional hazards model and reported as HR (95% confidence interval (CI)).

## 3 RESULTS

As previously reported, the primary analysis demonstrated no difference in 24‐month DFS in the ITT population comparing vaccine versus placebo (38.5% vs. 27.0%, HR 0.97 (95% CI 0.63–1.57), *p* = 0.974) but demonstrated a significantly improved 24‐month DFS in the PT population (62.9% vs. 34.8%, HR 0.52 (95% CI 0.27–0.98), *p* = 0.041) with a median follow‐up of 19.5 months.^14^ Median follow‐up ranged from 19.0 to 19.8 months for each of the analyzed subgroups, except for those who received CPI, which was 16.0 months.

### 3.1 Stage

Among patients with stage III resected melanoma (80 vaccine, 32 control), there were no clinical, pathologic, or treatment differences between groups (Table 1A). There was no difference in 24 month DFS between vaccine and placebo in the ITT population (36.9% vs. 35.5%,HR 0.80 (95% CI 0.46–1.38), *p* = 0.414) or in the PT population (59.7% vs. 44.0%, HR 0.73 (95% CI 0.35–1.55), *p* = 0.410).

**TABLE 1:** Clinical, pathologic, and treatment data for each subgroup examined, vaccine versus placebo. Proportions are compared with chi‐square test within each subgroup category

1A							
		Stage III			Stage IV		
		TLPLDC (%)	Placebo (%)	*p*‐value	TLPLDC (%)	Placebo (%)	*p*‐value
	*n*	80 (71)	32 (29)		23 (72)	28 (9)	
Age	Median	65.0	58.1	0.147	69.3	59.4	0.071
Margins	Positive	23 (21)	12 (11)	0.507	3 (9)	1 (3)	0.912
	Negative	33 (29)	14 (13)		13 (41)	5 (16)	
	NA	24 (21)	6 (5)		7 (22)	3 (9)	
BRAF Mutation	Yes	34 (30)	11 (10)	0.351	14 (44)	5 (16)	0.914
	No	33 (29)	12 (11)		5 (16)	2 (6)	
	NA	13 (12)	9 (8)		4 (13)	2 (6)	
Immunotherapy	Yes	29 (26)	9 (8)	0.412	12 (38)	6 (19)	0.457
	No	51 (46)	23 (21)		11 (34)	3 (9)	
CPI	Yes	21 (19)	5 (4)	0.229	12 (38)	4 (13)	0.694
	No	59 (53)	27 (24)		11 (34)	5 (16)	

1B							
		Immunotherapy			No Immunotherapy		
		TLPLDC (%)	Placebo (%)	*p*‐value	TLPLDC (%)	Placebo (%)	*p*‐value
	*n*	41 (73)	15 (27)		62 (70)	26 (30)	
Age	Median	61.3	60.3	0.287	66.1	56.7	0.070
Margins	Positive	7 (13)	5 (9)	0.467	19 (22)	8 (9)	0.820
	Negative	20 (36)	13 (7)		26 (30)	12 (14)	
	NA	14 (25)	3 (5)		17 (19)	6 (7)	
BRAF Mutation	Yes	25 (45)	7 (13)	0.341	23 (26)	9 (10)	0.627
	No	10 (18)	4 (7)		28 (32)	10 (11)	
	NA	6 (11)	4 (7)		11 (13)	7 (8)	
Stage	III	29 (52)	9 (16)	0.446	51 (58)	23 (26)	0.468
	IV	12 (21)	6 (11)		11 (13)	3 (3)	
CPI	Yes	33 (59)	9 (16)	0.117	0	0	0.000
	No	8 (14)	6 (11)		62 (70)	26 (30)	

1C							
		CPI			No CPI		
		TLPLDC (%)	Placebo (%)	*p*‐value	TLPLDC (%)	Placebo (%)	*p*‐value
	*n*	33 (79)	9 (21)		70 (69)	32 (31)	
Age	Median	63.4	58.7	0.236	65.6	58.2	0.083
Margins	Positive	4 (10)	4 (10)	0.820	22 (22)	9 (9)	0.467
	Negative	17 (40)	3 (7)		29 (28)	16 (16)	
	NA	12 (29)	2 (5)		19 (19)	7 (7)	
BRAF Mutation	Yes	20 (48)	6 (14)	0.627	28 (27)	10 (10)	0.341
	No	8 (19)	2 (5)		30 (29)	12 (12)	
	NA	5 (12)	1 (2)		12 (12)	10 (10)	
Stage	III	21 (50)	5 (12)	0.468	59 (58)	27 (26)	0.446
	IV	12 (29)	4 (10)		11 (11)	5 (5)	

1D							
		Primary			Recurrent		
		TLPLDC (%)	Placebo (%)	*p*‐value	TLPLDC (%)	Placebo (%)	*p*‐value
	*n*	73 (76)	23 (24)		30	18	
Age	Median	65.7	57.4	0.076	64.7	59.0	0.371
Margins	Positive	14 (15)	7 (7)	0.643	12 (25)	6 (13)	0.612
	Negative	35 (36)	10 (10)		11 (23)	9 (19)	
	NA	24 (25)	6 (6)		7 (15)	3 (6)	
BRAF Mutation	Yes	36 (38)	8 (8)	0.281	12 (25)	8 (17)	0.948
	No	25 (26)	7 (7)		13 (27)	7 (15)	
	NA	12 (13)	8 (8)		5 (10)	3 (6)	
Stage	III	54 (56)	19 (20)	0.397	26 (54)	13 (27)	0.215
	IV	19 (20)	4 (4)		4 (8)	5 (10)	
Immunotherapy	Yes	25 (26)	8 (8)	0.962	16 (33)	7 (15)	0.332
	No	48 (50)	15 (16)		14 (29)	11 (23)	
CPI	Yes	21 (22)	3 (3)	0.129	12 (25)	6 (13)	0.644
	No	52 (54)	20 (21)		18 (38)	12 (25)	

**FIGURE 1:**
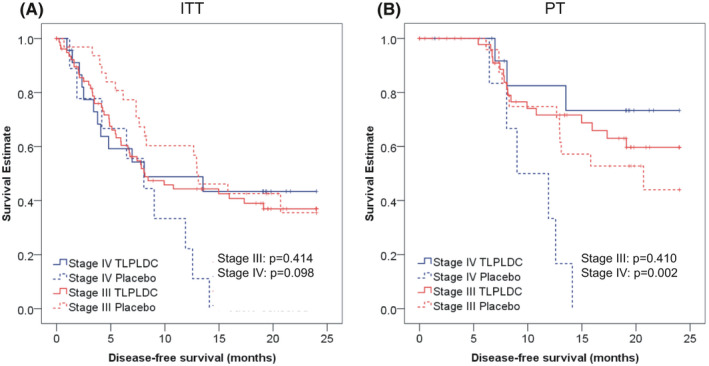
Kaplan‐Meier curves demonstrating 24‐month DFS stratified by stage for the ITT analysis (1A, left) and PT analysis (1B, right). Log rank p‐values are displayed within each figure

Among patients with stage IV resected melanoma (23 vaccine, 9 control), 24‐month DFS in the ITT population was 43.4% in the vaccine group and 0% in the placebo group (43.4% vs. 0%, HR 0.48 (95% CI 0.20–1.16), *p* = 0.098). In the PT population, there was a significant improvement in 24‐month DFS with vaccine vs. placebo (73.3% vs. 0%, HR 0.14 (95% CI 0.03–0.60), *p* = 0.002, Figure 1). Stage IV patients, regardless of treatment arm, were more likely than stage III patients to have received CPI (50% of stage IV patients vs. 26% of stage III patients, *p* = 0.003).

### 3.2 Immunotherapy

The number of patients receiving immunotherapy is summarized in Table 2. There were no clinical, pathologic, or treatment differences between groups when stratified for receiving immunotherapy (Table 1B). There was no difference between vaccine and control groups in the proportion of patients who received immunotherapy. Immunotherapy included IL‐2, IFN‐α, and/or CPI.

**TABLE 2:** Proportion of subjects receiving various types of immunotherapy. In terms of immunotherapy, in addition to interferon, interleukin, and CPI, one TLPLDC patient received TVEC and one placebo patient received BCG

	TLPLDC (%)	Placebo (%)	*p*‐value
Interferon	6 (6)	3 (7)	0.709
Interleukin	3 (3)	2 (5)	0.666
CPI	33 (32)	9 (22)	0.229

**FIGURE 2:**
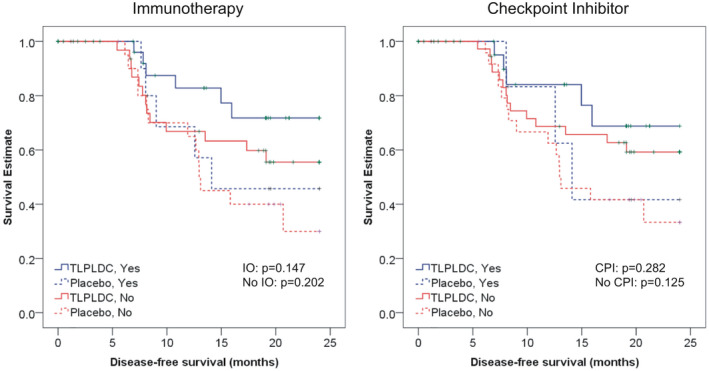
Kaplan‐Meier curves demonstrating 24‐month DFS in the PT analysis. Each arm is grouped by treatment arm (TLPLDC vs. placebo) and whether the patient received immunotherapy (IO) (yes/no, left) or checkpoint inhibitor (yes/no, right)

Among patients who did not receive any form of immunotherapy prior to randomization (62 vaccine, 26 control), there was no difference in 24‐month DFS between vaccine and placebo in the ITT population (30.3% vs. 24.2%, HR 1.22 (95% CI 0.70–2.14), *p* = 0.48) or in the PT population (55.5% vs. 30.0%, HR 0.61 (95% CI 0.28–1.32), *p* = 0.202, Figure 2A).

Among patients who did receive some form of immunotherapy prior to randomization (41 vaccine, 15 control), there was no difference in 24‐month DFS between vaccine and placebo in the ITT population (51.3% vs. 32.7%, HR 0.71 (95% CI 0.32–1.60), *p* = 0.411) or in the PT population (71.8% vs. 45.7%, HR 0.43 (95% CI 0.13–1.40), *p* = 0.147, Figure 2A). However, compared to the patients who did not receive immunotherapy, there was greater separation in the KM curves between the vaccine and placebo groups in both ITT and PT populations.

### 3.3 Checkpoint inhibitor

There were no clinical, pathologic, or treatment differences between groups when stratified by receipt of CPI (Table 1C). Between vaccine and control groups irrespective of stage, there was no difference in the proportion of patients receiving CPI. Of the 42 patients who received CPI, six received combination therapy with a CTLA‐4 inhibitor and a PD‐1 inhibitor (5 vaccine, 1 control), 19 received a CTLA‐4 inhibitor alone (13 vaccine, 6 control), and 17 received a PD‐1 inhibitor alone (15 vaccine, 2 control).

Among patients who were not treated with CPI (70 vaccine, 32 control), there was no significant difference in 24‐month DFS between vaccine and control in the ITT population (33.4% vs. 26.0%, HR 1.11 (95% CI 0.55–1.86), *p* = 0.701) or in the PT population (59.2% vs. 33.3%, HR 0.57 (0.27–1.18), *p* = 0.125, Figure 2B).

Among patients who were treated concurrently with CPI (33 vaccine, 9 control), there was no significant difference in 24‐month DFS between vaccine and control in the ITT population (49.3% vs. 31.3%, HR 0.82 (95% CI 0.30–2.29), *p* = 0.710) or in the PT population (68.8% vs. 41.7%, HR 0.46 (95% CI 0.11–1.95), *p* = 0.282, Figure 2B). However, when compared to patients who did not receive CPI, there was greater separation in the KM curves between the vaccine + CPI versus placebo + CPI groups in both ITT and PT populations.

### 3.4 Primary and recurrent disease

Patients in the ITT cohort were divided into a recurrent disease group (n = 48) and a primary disease group (n = 96). There were no differences between groups in stage, age, margin status, BRAF mutation, or use of concurrent immunotherapy including CPI (Table 1D).

**FIGURE 3:**
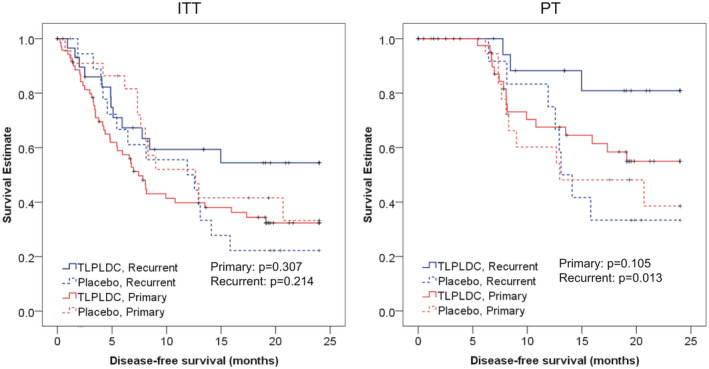
Kaplan‐Meier curves demonstrating 24‐month DFS stratified by Disease‐Free Interval, characterized as Recurrent or Primary disease, where Primary disease included patients without prior melanoma and those with a disease‐free interval of less than 3 months. Recurrent disease included patients with DFI >3 months

Among patients in the resected primary disease group, there was no difference in 24‐month DFS in the ITT population between vaccine and placebo (32.4% vs. 33.3%, *p* = 0.307, Figure 3A) or in the PT population (54.4% vs. 22.2%, *p* = 0.105, Figure 3B).

Among patients in the resected recurrent disease group, 24‐month DFS in the ITT population was 52.6% in the vaccine group versus 23.5% in the placebo group (*p* = 0.214, Figure 3A). In the PT population, there was significantly improved 24‐month DFS in the vaccine group (80.9% vs. 33.3%, *p*=0.013, Figure 3B).

### 3.5 Dose response

**FIGURE 4:**
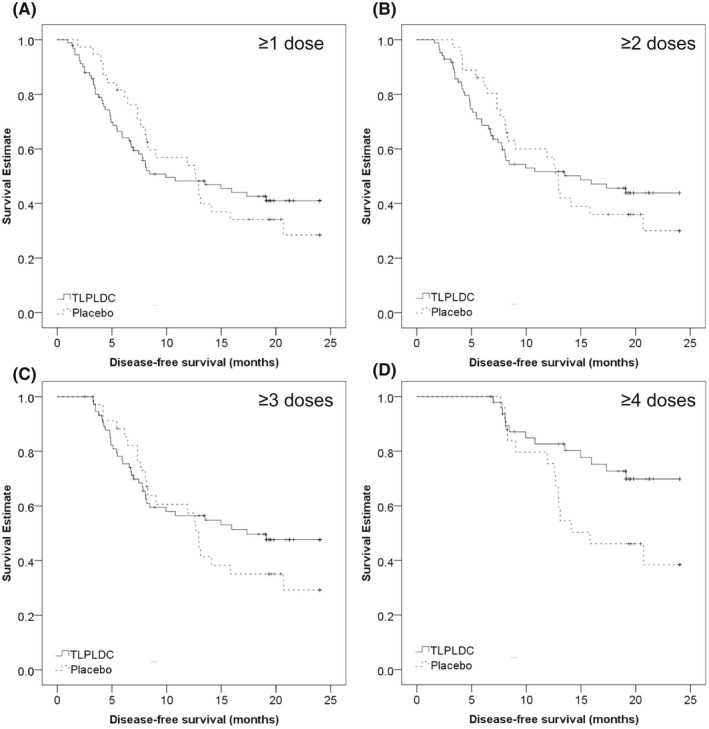
Kaplan‐Meier curves demonstrating 24‐month DFS comparing TLPLDC vs. placebo for patients receiving ≥1 dose (A) top left, ≥2 doses (B) top right, ≥3 doses (C) bottom left, and ≥4 doses (D) bottom right

An exploratory analysis was conducted to evaluate whether there is a relationship between number of vaccine doses and clinical outcome. As previously reported, there was a significant difference in 24‐month DFS between TLPLDC and placebo in patients receiving the entire 4 dose PVS, which is equivalent to the PT group (69.8% vs. 38.4%, *p* = 0.024, Figure 4A).

There was no significant difference in 24‐month DFS in subgroups receiving≥3 doses (47.7% vs. 29.2%, *p* = 0.380, Figure 4B), ≥2 doses (43.9% vs. 30.0%, *p* = 0.784, Figure 4C), or ≥1 dose (41.0% vs. 28.4%, *p* = 0.904, Figure 4D). However, the separation in KM curves increased with the number of doses received.

## 4 DISCUSSION

We have previously reported the results of our primary analysis of this phase IIb trial, which demonstrated that the vaccine is safe, and improved DFS in patients who were able to complete the PVS.^14^ In this study, we examined the variable benefit of vaccination across multiple subgroups. First, we found that the benefit of vaccination was essentially limited to patients who completed the PVS, and did not extend to patients who received fewer than four doses of the vaccine. Next, there was no difference in 24‐month DFS with vaccination in stage III patients, but the TLPLDC significantly improved 24‐month DFS in the PT analysis of stage IV patients. Analyzing patients by concurrent immunotherapy and vaccination, there was no significant difference in 24‐month DFS when stratified by use of immunotherapy or CPI; however, the DFS was highest in patients receiving both vaccine and immunotherapy/CPI. Finally, there was improved 24‐month DFS with vaccination in patients who enrolled with recurrent melanoma and completed the PVS, but not in patients enrolled with primary stage III/IV disease.

The trial's primary analysis demonstrated a benefit to the TLPLDC vaccine in the PT analysis, which was limited to patients who were able to complete the PVS without early recurrence.^14^ As part of the current study, we looked at patients who received partial therapy. The vaccine had no significant benefit in patients receiving one, two, or three doses; all benefit was derived by those able to complete the PVS. However, there was an increasing DFS based on the number of doses received. There are two possible explanations for this finding. First, there is some evidence that immunotherapy requires multiple doses to create an effective anti‐tumor immune response, and it is possible this is particularly true for cancer vaccines. Thus, there may be a minimum therapeutic threshold to create an effective response. Second, it is important to note the biology of patients who recurred within six months of enrollment, which is likely a surrogate for more aggressive disease biology. Patients who received up to three doses of the vaccine but recurred prior to their six‐month visit did not benefit from vaccination, again likely because this early recurrence was a marker of aggressive disease biology. It has been demonstrated repeatedly that patients with more aggressive biology are less likely to derive benefit from monotherapy with a cancer vaccine.^12^, ^14^ Regardless, we believe the PT analysis is indicative of the true response to vaccination in a properly selected patient population. Thus, we examined both the ITT and PT populations for the remainder of our subgroup analyses.

Despite the general theme that vaccines tend to work better for patients with less aggressive disease,^15^ our pre‐specified analysis revealed that stage IV patients had a significant benefit from vaccination, while stage III patients did not, at the time of this analysis. While this finding may be paradoxical, it may be related to either patient selection and/or timing of this analysis. We included only stage IV patients who underwent resection of metastatic disease as part of standard of care therapy. These highly selected patients, despite advanced stage, likely have more biologically indolent disease than the average patient with metastatic disease, and potentially even more indolent than some patients with stage III disease. Indeed, it is likely the disease biology, more than the clinical stage, that determines response to vaccine therapy. Additionally, given the higher risk and shorter time to recurrence in stage IV resected patients compared to stage III patients, we may be seeing earlier benefit in the stage IV patients, and we may not see any potential benefit of the vaccine until we analyze the trial with longer follow‐up at the secondary endpoint of 36 months.

The final two pre‐specified subgroups examined were patients receiving immunotherapy, and those receiving CPI specifically, as part of their standard therapy. Multiple early studies suggest benefit of combination immunotherapy, including synergy between vaccines and CPIs, with the vaccine stimulating a T cell response, and CPIs serving to enhance the lifespan and function of these effector T cells.^16^, ^17^, ^18^, ^19^ There are several ongoing trials of combination immunotherapy, which is indicative of the promise of this strategy. In our study, there was no significant benefit to vaccination within the immunotherapy or CPI subsets; however, the highest observed DFS rates were seen in the combination treated patients. Unfortunately, our subgroup analyses were significantly limited by small sample size, making the chances of a type II error significant. There was a numerical benefit to combination of vaccine and CPI compared to CPI and placebo in both ITT and PT analysis, which may be verified if studied in a larger group. These findings will be evaluated in future trials with improved patient selection and larger sample sizes.

In an exploratory subgroup analysis of patients with primary or recurrent disease at presentation, there was significant benefit from the vaccine in patients with resected recurrent melanoma. This differential benefit is again likely related to disease biology. Patients who enrolled with recurrent disease may have initially presented at a lower stage, whereas patients enrolled with primary disease, by definition, presented with stage III or IV disease. Thus, despite being enrolled for stage III/IV disease, these patients with recurrent disease may have had a significant disease‐free interval prior to recurrence. These patients may have more indolent disease biology and a less immunosuppressive tumor microenvironment, where monotherapy with a cancer vaccine is thought to be more effective. Further work to better elucidate this mechanism as it relates to the TLPLDC vaccine is ongoing.

This study has a number of limitations. First, the sample size of each subgroup is small. While this is unavoidable and in the nature of such an analysis, it does limit the power of each individual analysis and raises the question of type II error in the conclusions drawn from each subgroup. Next, while most subgroups were pre‐specified in the protocol, some were exploratory, limiting any substantial conclusions from these groups. These exploratory subgroup analyses are, however, hypothesis‐generating, and are not intended to make definitive conclusions. Additionally, the use of immunotherapy, including CPI, was not randomized; it was instead given at the discretion of the treating oncologist. Furthermore, use of these therapies changed drastically during the time in which this study was conducted. Finally, the lack of correlative immunologic data makes it difficult to analyze the various hypotheses we propose to explain our findings in this study. Unfortunately, assessing tumor response or immune infiltration is not possible as all patients were disease‐free at the time of enrollment. Similarly, peripheral immunologic assays are not practical given the personalized nature of the vaccine, as we do not know each patient's unique tumor antigens/neo‐antigens, and no single assay would be reliable across many patients. Despite these limitations, data regarding variable benefit in these subgroups remains valuable and will help define future trials of the TLPLDC vaccine.

Given the promising findings of our trial thus far, further study of TLPLDC in a well‐selected patient population is warranted. As previously discussed, CPI are currently a standard option for systemic adjuvant treatment in stage III and stage IV melanoma. Therefore, the phase III trial of TLPLDC will compare TLPLDC + PD‐1 inhibitor versus placebo + PD‐1 inhibitor alone in patients with resected stage IV melanoma. The aim of the study will be to determine whether the addition of TLPLDC, a personalized vaccine, increases the efficacy of the PD‐1 inhibitor in preventing melanoma recurrence.

## 5 CONCLUSION

In conclusion, this subgroup analysis demonstrates that the TLPLDC vaccine is most effective at preventing recurrence in patients with resected stage IV melanoma who received at least the six‐month PVS. Conclusions about synergy between TLPLDC and other forms of immunotherapy are limited by sample size and randomization but appear promising. Based on these findings, and the widespread application of CPI as first‐line adjuvant therapy following surgery for advanced melanoma, the efficacy of the TLPLDC vaccine will be further studied in a phase III trial of TLPLDC versus placebo in combination with CPI.

## CONFLICT OF INTEREST

Dr. Faries is an advisor for Bristol Myers‐Squibb, Sanofi, Array Bioscience, and Pulse Bioscience. Dr. Hyngstrom has received funding from Castle Biosciences and Amgen. Dr. Berger has received funding from Castle Biosciences and Cardinal Health. Ms Park and Ms Sloan are employed by Orbis Health Solutions. Dr. Wagner is employed by Orbis Health Solutions and Perseus PCI and has received funding from Elios Therapeutics. Dr. Peoples is employed by Orbis Health Solutions and Cancer Insight; is a consultant for Rapamycin Holdings, Heat Biologics, Abexxa Biologics, and Pelican Therapeutics; and has received funding from the above as well as Sellas Life Sciences and Genentech.

## ETHICAL APPROVAL STATEMENT

This clinical trial was approved by the Western Institutional Review Board® (WIRB) under Protocol #20141932 and was conducted in accordance with federal law and accepted international standards. The findings were monitored throughout the study by a Data Safety Monitoring Board (DSMB).
